# Spiral Bronchial Rupture in a Young Trauma Patient: Critical Role of Bronchoscopy and Surgical Precision

**DOI:** 10.7759/cureus.78351

**Published:** 2025-02-01

**Authors:** Vasileios Leivaditis, Vasileios Karamouzos, Diamanto N Aretha, Virginia Mplani, Athanasios Papatriantafyllou, Christos Prokakis, Foteini Fligou, Efstratios N Koletsis

**Affiliations:** 1 Cardiothoracic and Vascular Surgery, Westpfalz-Klinikum, Kaiserslautern, DEU; 2 Intensive Care Unit, General University Hospital of Patras, Patras, GRC; 3 Anesthesiology and Critical Care, Genral University Hospital of Patras, Patras, GRC; 4 Cardiothoracic Surgery, General University Hospital of Patras, Patras, GRC

**Keywords:** airway management, bronchial stump closure, fiberoptic bronchoscopy, pneumonectomy, thoracic trauma, tracheobronchial injury, traumatic bronchial rupture

## Abstract

Traumatic tracheobronchial injuries (TTBIs) are rare but potentially life-threatening complications of high-energy thoracic trauma, most commonly associated with road traffic accidents. These injuries demand prompt diagnosis and a multidisciplinary approach for effective management. We present the case of a 16-year-old male patient who sustained a spiral rupture of the right main bronchus following a motorcycle accident. Upon presentation to the emergency department (ED), the patient demonstrated severe hypoxemia and respiratory acidosis. Imaging confirmed the presence of a pneumothorax, necessitating the placement of bilateral chest tubes. Despite the intervention, a persistent air leak was observed. Fiberoptic bronchoscopy was used to confirm the diagnosis and stabilize the patient by isolating the unaffected left lung. Definitive surgical management via right thoracotomy revealed a complex spiral lesion in the right main bronchus, which was deemed irreparable. A right pneumonectomy was performed, and the bronchial stump was secured with 4-0 pledgeted sutures reinforced with fibrin glue. The patient’s postoperative course was uneventful, and he was discharged in good health 15 days later. Further follow-up demonstrated excellent health and no complications. This case highlights the critical role of fiberoptic bronchoscopy in the diagnosis and initial stabilization of TTBIs and emphasizes the importance of meticulous surgical techniques for managing complex bronchial injuries. The combination of prompt airway management and careful surgical planning ensured a successful outcome, even in the context of a severe, life-threatening, and technically challenging injury.

## Introduction

Traumatic injuries to the tracheobronchial tree (TBT) are rare but life-threatening clinical entities that require prompt recognition and management to avoid severe morbidity or mortality. These injuries typically result from high-energy trauma, with road traffic accidents being the most common cause [1-3]. The rigid thoracic anatomy in adults predisposes them to such injuries, whereas in children, the elastic nature of the thoracic cage provides some degree of protection. Nonetheless, when tracheobronchial injuries (TTBIs) occur in pediatric or adolescent patients, the consequences can be catastrophic due to their smaller airway diameter and limited physiological reserves [2].

The majority of TTBIs involve the main bronchi within 2.5 cm of the carina, as this region is anatomically vulnerable to compression against the vertebral column during rapid deceleration injuries. Left main bronchus injuries are relatively less frequent due to the protective presence of the aortic arch. Common clinical presentations of TTBIs include respiratory distress, subcutaneous emphysema, hemoptysis, pneumothorax, and air leakage that is refractory to standard interventions such as thoracostomy tube placement [1,3].

Diagnosing TBT injuries is challenging, as these injuries are often masked by other traumatic lesions in polytrauma patients. A high index of suspicion is critical, particularly in patients with persistent air leaks, respiratory compromise, or radiological findings such as pneumothorax or collapsed lung that fail to resolve after conventional treatment. Fiberoptic bronchoscopy is the diagnostic modality of choice, as it allows direct visualization of the injury while simultaneously enabling therapeutic interventions such as selective ventilation of the unaffected lung [4-6].

Definitive management of TTBIs typically requires surgical intervention. Primary repair of the airway is preferred when feasible, but the nature and extent of the injury may necessitate more extensive procedures, such as lobectomy or pneumonectomy. Postoperative care and follow-up are essential to monitor for complications such as bronchial stump dehiscence, infection, or long-term respiratory impairment [1,7].

This report presents the case of a 16-year-old male patient who sustained a near-fatal rupture of the right main bronchus following a motorcycle accident. The injury was identified using fiberoptic bronchoscopy, which also played a pivotal role in the initial stabilization of the patient. Despite efforts at primary repair, the severity and spiral nature of the bronchial lesion necessitated a right pneumonectomy. The case emphasizes the importance of a multidisciplinary approach and highlights the critical role of bronchoscopy in the diagnosis and management of such rare injuries.

## Case presentation

A 16-year-old male patient, previously healthy, was transported to the emergency department (ED) following a high-energy motorcycle accident. During transportation by ambulance, he reported severe chest pain and progressive dyspnea. On arrival at the ER, the patient was agitated, confused, and exhibited signs of severe hypoxemia, with arterial oxygen saturation markedly reduced despite oxygen supplementation. The patient was unstable, and an emergency intubation was performed.

Initial examination and investigations

Clinical examination revealed tachypnea (31/min), diminished breath sounds bilaterally on auscultation, and subcutaneous emphysema, particularly in the upper thoracic region. A chest X-ray (CXR) demonstrated bilateral pneumothoraces, prompting the immediate insertion of bilateral chest tubes.

**Figure 1:**
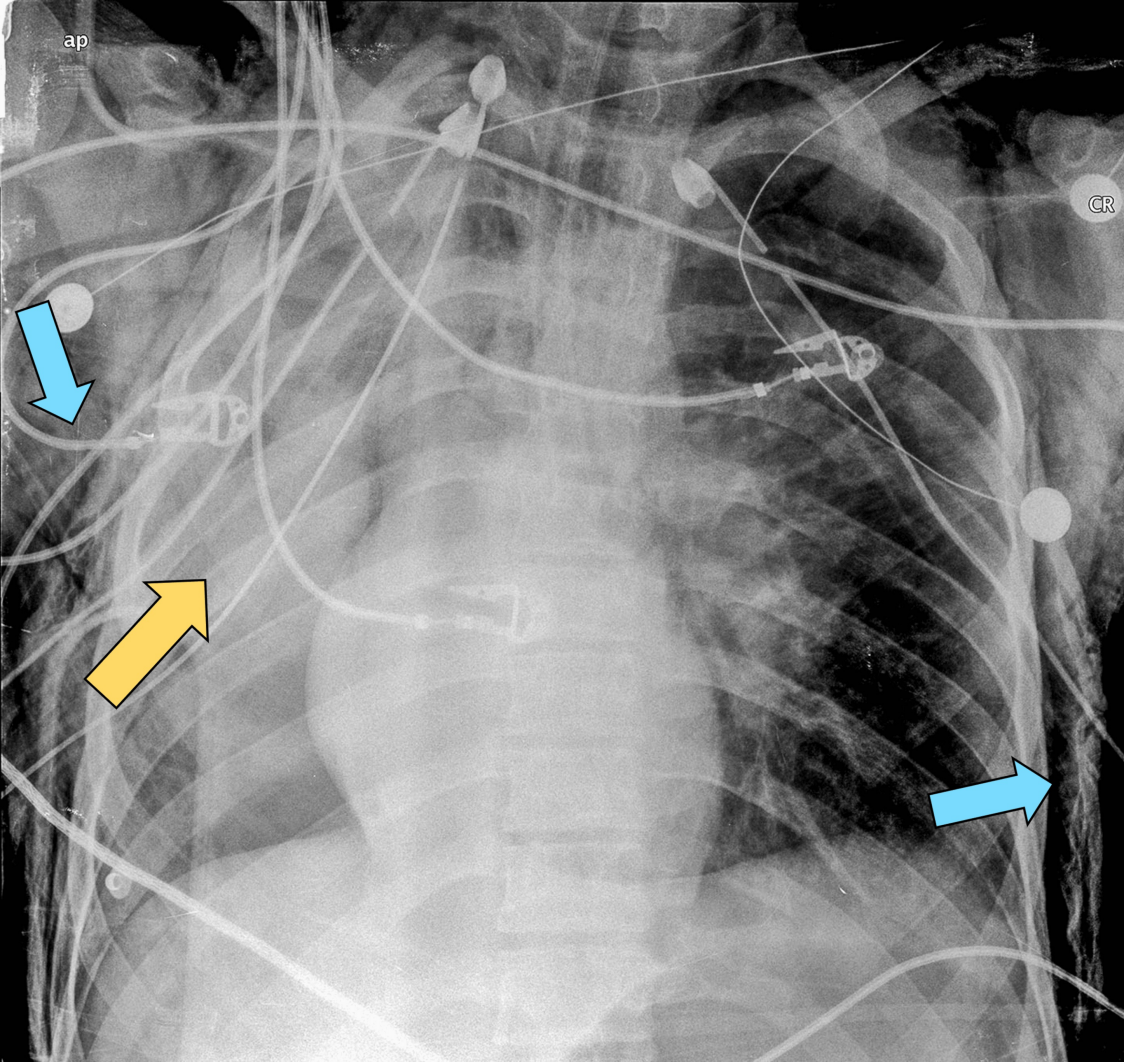
Chest X-ray showing the persistent collapse of the right lung (yellow arrow) and subcutaneous emphysema (blue arrows) despite the placement of bilateral chest tubes.

Despite this intervention, a persistent air leak was observed from the right chest tube. A repeat CXR revealed a persistent complete collapse of the right lung (Figure 1). Initial arterial blood gas (ABG) analysis showed severe hypoxemia (P/F ratio <100) and significant respiratory acidosis, with a partial pressure of carbon dioxide (PCO₂) of 90 mmHg and a pH of 7.2. Given the persistent air leak and severe hypoxemia, a high suspicion for TTBI was raised.

Emergency bronchoscopy and airway management

To confirm the diagnosis and manage the patient’s deteriorating respiratory status, an emergency fiberoptic bronchoscopy was performed. The procedure revealed a significant rupture of the right main bronchus approximately 2 cm distal to the carina. The lesion was spiral in nature, extending circumferentially and causing a significant disruption in the airway continuity.

The endotracheal tube (ETT) cuff was deflated, and the tube was advanced under direct visualization into the left main bronchus to isolate the healthy lung. Once the ETT cuff was reinflated, the persistent air leak ceased, and ABG parameters began to improve.

**Figure 2:**
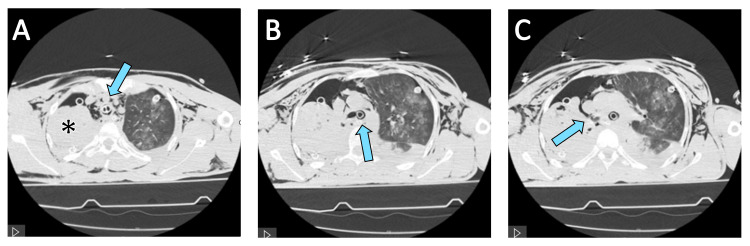
Computed tomography (CT) images illustrating: (A) complete collapse of the right lung (asterisk) and the position of the double-lumen endotracheal tube within the trachea (arrow); (B) the course of the double-lumen tube advanced into the left main bronchus (arrow), enabling selective ventilation of the left lung; (C) the suspected rupture site of the right main bronchus (arrow), corresponding to the spiral lesion, was identified intraoperatively.

Using a bougie catheter as a guide, the single-lumen ETT was replaced with a double-lumen tube to facilitate selective ventilation of the left lung. A full-body computed tomography (CT) scan was subsequently performed, revealing hemorrhagic brain contusions without significant intraparenchymal bleeding. A limited subarachnoid hemorrhagic effusion was also identified. In the thoracic cavity, the CT scan demonstrated total atelectasis of the right lung, a defect in the anterior wall of the right main bronchus, and extensive subcutaneous emphysema. Additionally, contusions were observed in the left lung parenchyma (Figure 2). After stabilization, the patient was promptly transferred to the operating room for definitive surgical intervention.

Surgical findings and management

**Figure 3:**
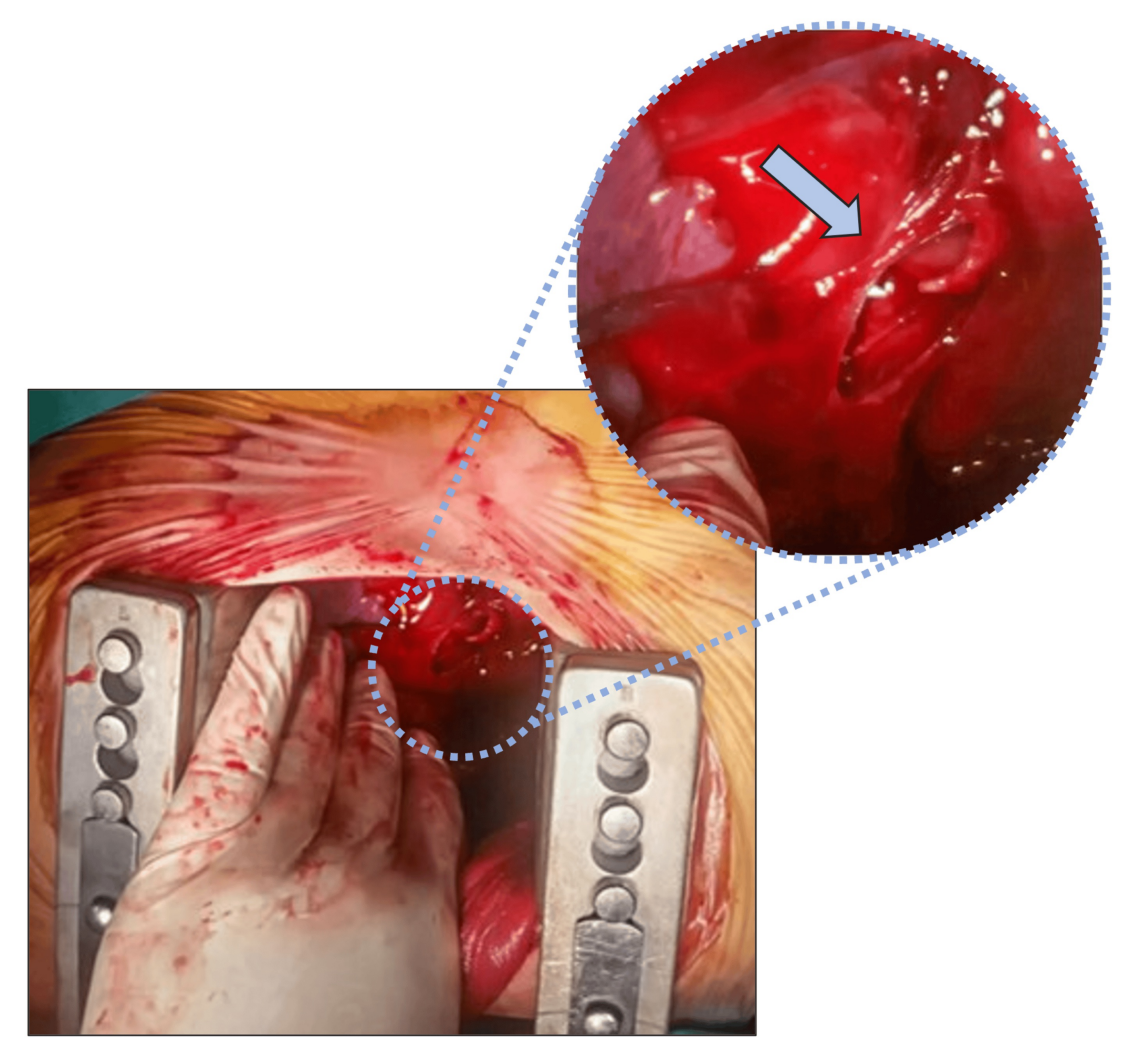
Intraoperative image depicting the spiral rupture of the right main bronchus (arrow).

A right thoracotomy was performed, revealing a spiral, full-thickness rupture of the right main bronchus extending approximately 2 cm from the carina to the bronchial branch of the upper lobe. The rupture exhibited both longitudinal and transverse components, resulting in subtotal dissection of the main bronchus (Figure 3). The bronchus to the right upper lobe was also significantly affected, with subtotal dissection observed. Additionally, the rupture of the membranous portion of the bronchial tree extended further into the intermediate bronchus. A severe laceration of the pulmonary parenchyma in the upper lobe was also identified. Despite attempts to repair the extensive damage, the spiral configuration and the severity of the injuries rendered primary repair technically unfeasible.

As a result, a right pneumonectomy was performed. The bronchial stump was meticulously closed using 4-0 pledgeted Prolene polypropylene sutures (Ethicon Inc., Cornelia, Georgia) to ensure a secure and robust seal. To further reinforce the closure and prevent air leaks, fibrin glue was applied along the suture line. Additionally, the stump was covered with an intercostal muscle flap, which had been carefully prepared at the start of the thoracotomy. Hemostasis was thoroughly achieved, and the patient was subsequently transferred to the intensive care unit (ICU) for close postoperative monitoring.

Postoperative course and follow-up

In the ICU, the patient remained hemodynamically stable, with progressive improvement in respiratory function. Serial ABG analyses showed normalization of oxygenation and resolution of hypercapnia. However, due to challenges with weaning from mechanical ventilation, a percutaneous tracheotomy was performed on the fifth postoperative day, allowing for gradual and successful weaning from the ventilator. Follow-up CT scans confirmed the complete resolution of the brain injuries.

**Figure 4:**
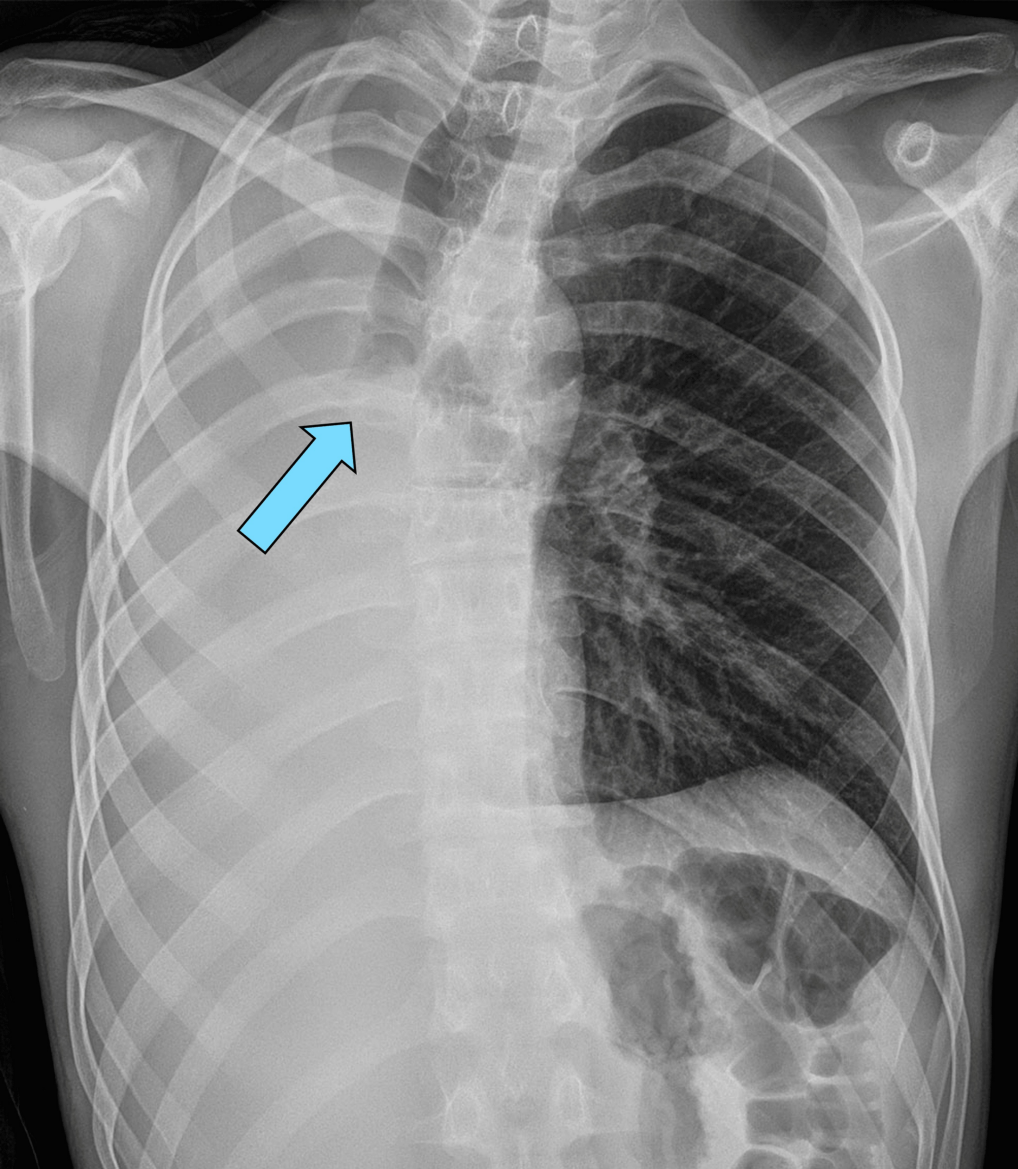
Postoperative chest X-ray demonstrating normal filling of the right pleural cavity following pneumonectomy. The image also shows the intact and well-sealed right bronchial stump (arrow).

On the first postoperative day, the patient developed a fever and elevated inflammatory markers (white blood cell (WBC): 16,700/μL; C- reactive protein (CRP): 186 mg/L). Perioperatively, blood cultures were obtained, which isolated *Klebsiella pneumoniae*, and an initial antibiotic regimen of meropenem and vancomycin was promptly started based on infectious disease consultation. One week later, new blood cultures revealed the presence of *Acinetobacter baumannii*, prompting a modification of the antibiotic regimen to include meropenem, linezolid, colistin, and tigecycline. The patient’s treatment and clinical progress were closely monitored during this period. Early mobilization and physiotherapy were introduced to support recovery and enhance physical function. A postoperative CXR demonstrated normal healing of the right pleural cavity, indicating favorable progress (Figure 4).

On postoperative day 22, the patient was transferred to a general ward in stable condition, asymptomatic, and free from any residual respiratory distress. He was discharged on postoperative day 34 in good overall health. Subsequent follow-up evaluations confirmed that the patient remained asymptomatic and in excellent health.

**Figure 5:**
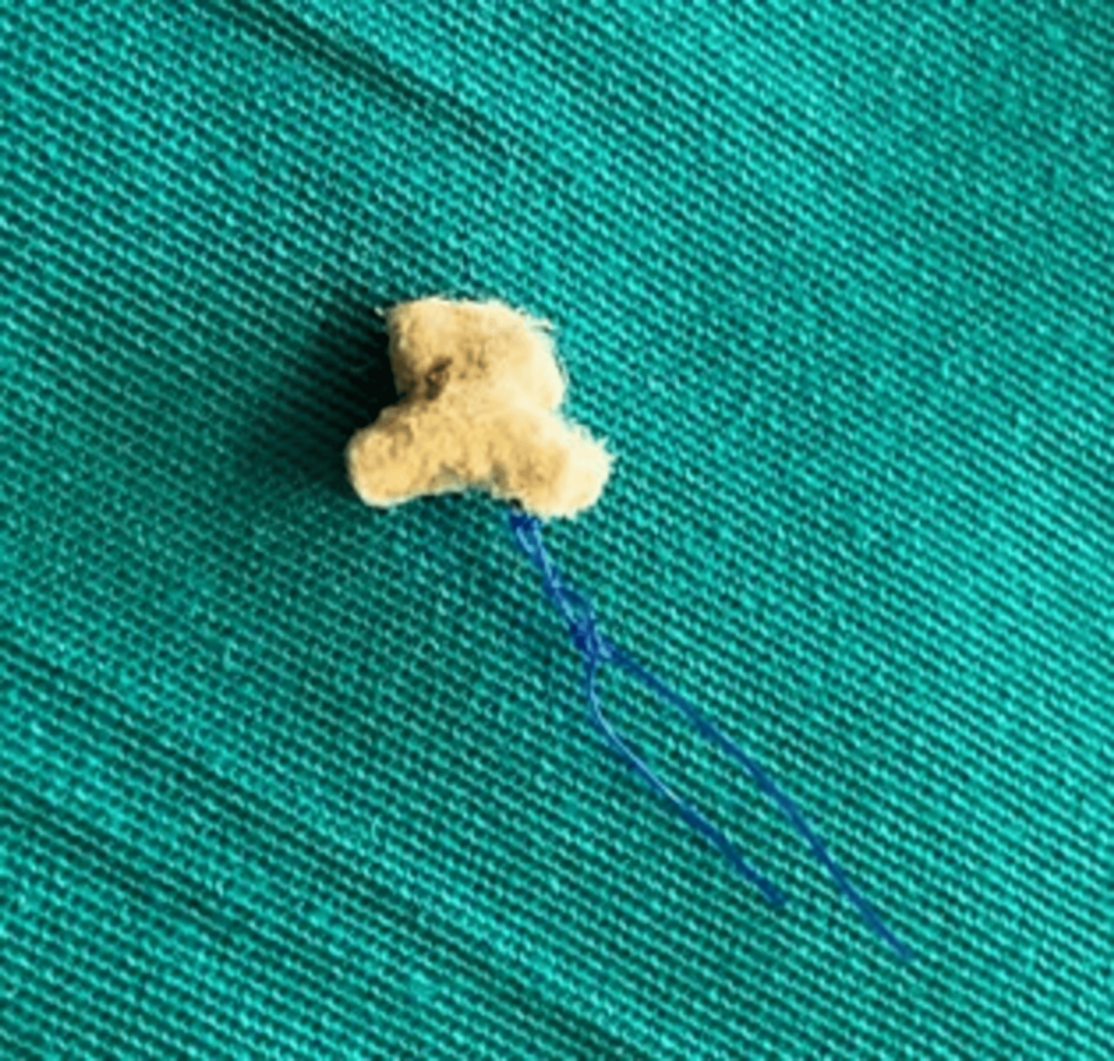
The pledgeted 4-0 Prolene suture which was expectorated by the patient one year after the operation.

Two years later, the patient was referred back to our department after expectorating a foreign body. Upon evaluation, the foreign body was identified as one of the sutures from the bronchial stump (Figure 5). A follow-up bronchoscopy was performed, which revealed a perfectly healed bronchial stump with no evidence of residual defects or complications. The patient continued to do well during subsequent follow-up appointments, and six years later, no further complications were noted.

## Discussion

Traumatic tracheobronchial injuries are rare clinical entities, with an estimated incidence of 0.05% to 2.5% in blunt thoracic trauma cases [1,3,8]. These injuries pose significant diagnostic and therapeutic challenges due to their rarity, varied presentations, and potential for rapid deterioration. Effective management hinges on early recognition, prompt stabilization, and a multidisciplinary approach to definitive care [5,6]. 

Airway management

Airway management is paramount in cases of suspected TTBIs, as patients often present with significant respiratory compromise [9]. In this case, the patient arrived in the emergency department with severe hypoxemia, respiratory acidosis, and a persistent air leak despite bilateral thoracostomy tube placement. These findings, coupled with radiographic evidence of a collapsed right lung, strongly suggested a major airway disruption.

Fiberoptic bronchoscopy is the gold standard for diagnosing TTBIs, as it allows direct visualization of the injury and facilitates therapeutic interventions [6,10]. In this case, bronchoscopy confirmed a spiral rupture of the right main bronchus approximately 2 cm from the carina. The use of bronchoscopy enabled selective isolation of the unaffected left lung by advancing the ETT into the left main bronchus. This maneuver not only stabilized the patient’s respiratory status but also prevented further air leaks and allowed time for definitive surgical planning.

Transitioning from a single-lumen to a double-lumen ETT, guided by a bougie catheter, ensured continued selective ventilation during surgery. This step was crucial in maintaining adequate oxygenation and minimizing the risk of ventilator-induced injury to the already compromised bronchial structures. The expertise required for such airway management highlights the importance of advanced bronchoscopic skills in managing TTBIs [11]. Additionally, this approach underscores the importance of a multidisciplinary team, including anesthesiologists, intensivists, and thoracic surgeons, in coordinating care [10].

Surgical management

Definitive surgical management of TTBIs is guided by the nature and extent of the injury [4,9]. In this case, the intraoperative findings revealed a spiral, full-thickness rupture of the right main bronchus. Despite initial attempts at primary repair, the complex geometry and extensive tissue damage rendered the bronchus irreparable. The spiral nature of the lesion, involving significant circumferential disruption, posed a substantial technical challenge, leaving pneumonectomy as the only viable option.

The incidence of lung resection due to traumatic injuries is rare, reported at 0.08%, with pneumonectomy being even less frequent at 0.01% among trauma patients [12]. Although modern surgical approaches, such as tractotomy and non-anatomic wedge resection, are often preferred, pneumonectomy remains necessary in cases of uncontrollable hilar hemorrhage or irreparable pulmonary and bronchial damage. However, the trauma rates range from 50% to 100%, as reported in various studies [12,13].

Pneumonectomy in a young patient is a significant undertaking, as it carries the risk of substantial short- and long-term morbidity. The first documented pneumonectomy was performed by Rudolph Nissen in 1931 on a 12-year-old girl with blunt pulmonary injuries [14]. Since then, trauma pneumonectomy has been utilized in critically injured patients presenting in extremis [12]. To optimize outcomes, meticulous attention was paid to securing the bronchial stump. A combination of 4-0 pledgeted sutures and fibrin glue was used to reinforce the stump and prevent postoperative air leaks or dehiscence. This approach reflects current best practices in thoracic surgery, where securing the bronchial stump is critical to avoid catastrophic complications such as bronchopleural fistula or empyema.

The postoperative course in this patient was uneventful, demonstrating the success of the surgical strategy. However, the long-term success of such interventions also depends on thorough follow-up. In this case, the patient remained asymptomatic until two years later, when he presented with the expectoration of a suture from the bronchial stump. A follow-up bronchoscopy at that time confirmed complete healing of the stump with no evidence of residual defects, underscoring the efficacy of the initial surgical approach. Trauma pneumonectomy remains a procedure with significant morbidity, even with modern advancements, and should be performed only in carefully selected patients [13,15].

## Conclusions

The successful management of this case highlights the importance of a structured and multidisciplinary approach to TTBIs. This case is particularly notable for the spiral nature of the bronchial rupture, which posed unique challenges for surgical repair and required precise coordination between bronchoscopic and surgical interventions. The thorough application of advanced bronchoscopic techniques facilitated accurate diagnosis and intraoperative guidance, while the surgical team adapted techniques to address the complex anatomy and extent of the injury. Furthermore, the decision to proceed with pneumonectomy in a young patient highlighted the balance of risk and benefit in extreme cases, with a favorable long-term outcome achieved through rigorous follow-up. This case provides valuable lessons for managing similar injuries, advocating for further exploration of surgical strategies and long-term outcomes in young patients undergoing major thoracic procedures.
